# P-Ergonomics Platform: Toward Precise, Pervasive, and Personalized Ergonomics using Wearable Sensors and Edge Computing

**DOI:** 10.3390/s19051225

**Published:** 2019-03-11

**Authors:** Mario Vega-Barbas, Jose A. Diaz-Olivares, Ke Lu, Mikael Forsman, Fernando Seoane, Farhad Abtahi

**Affiliations:** 1 Institute of Environmental Medicine, Karolinska Institutet, Solnavägen 1, 17177 Solna, Sweden; ke.lu@ki.se (K.L.); mikael.forsman@ki.se (M.F.); 2 School of Engineering Sciences in Chemistry, Biotechnology and Health, KTH Royal Institute of Technology, Hälsovägen 11C, 14157 Huddinge, Sweden; jadiaz@kth.se; 3 Department of Clinical Science, Intervention and Technology, Karolinska Institutet, Hälsovägen 7, 14157 Huddinge, Sweden; fernando.seoane@ki.se; 4 Swedish School of Textiles, University of Borås, Allégatan 1, 50190 Borås, Sweden; 5 Departm and t of Biomedical Engineering, Karolinska University Hospital, 1, 17176 Solna, Sweden

**Keywords:** disease prevention, occupational healthcare, P-Ergonomics, precision ergonomics, musculoskeletal disorders, smart textiles, wearable sensors, wellbeing at work

## Abstract

Preventive healthcare has attracted much attention recently. Improving people’s lifestyles and promoting a healthy diet and wellbeing are important, but the importance of work-related diseases should not be undermined. Musculoskeletal disorders (MSDs) are among the most common work-related health problems. Ergonomists already assess MSD risk factors and suggest changes in workplaces. However, existing methods are mainly based on visual observations, which have a relatively low reliability and cover only part of the workday. These suggestions concern the overall workplace and the organization of work, but rarely includes individuals’ work techniques. In this work, we propose a precise and pervasive ergonomic platform for continuous risk assessment. The system collects data from wearable sensors, which are synchronized and processed by a mobile computing layer, from which exposure statistics and risk assessments may be drawn, and finally, are stored at the server layer for further analyses at both individual and group levels. The platform also enables continuous feedback to the worker to support behavioral changes. The deployed cloud platform in Amazon Web Services instances showed sufficient system flexibility to affordably fulfill requirements of small to medium enterprises, while it is expandable for larger corporations. The system usability scale of 76.6 indicates an acceptable grade of usability.

## 1. Introduction

Musculoskeletal disorders (MSDs) are still common in the working population, causing individual suffering and an economic burden for companies and societies [1,2]. Further, the challenge of an aging population—an increased percentage of elderly individuals in the overall population—has occurred for more than two decades and its consequences, e.g., increasing costs, a lack of healthcare personnel, and more complex combinations of chronic diseases [3], have emerged. For economic reasons, a longer life expectancy requires an increase in the retirement age. These increases have already occurred in some countries and are planned in others [4,5].

To facilitate a prolonged work life for the general population, a balance between work demands and human capabilities must be found, especially for blue-collar workers [5]. This is in line with suggested approaches for chronic disease management to reduce the healthcare burden [6].

Interventions aiming to reduce MSD risk factors at work are not new. Ergonomists and work psychologists have tried to identify risk factors at specific workplaces to design and suggest physical, organizational, and when relevant, behavioral changes to reduce the risks. Work psychologists may use self-reports, and ergonomists often use observation-based assessment tools to identify these risks [7,8]. Observation methods are easy to use, inexpensive, and widely accessible, but the inter- and intra-observer reliability of such methods are relatively poor [7,9,10]. There are also substantial inter-method differences in risk assessments [11]. Another source of variance in risk assessments is the short observation time; the ergonomist often observes a minor part of the workday, which is then supposed to represent the full day. There are also differences in anthropometrics and work techniques between different workers, so risk assessments should include several workers to increase reliability.

Popular ergonomic risk assessment methods are mostly based on evaluation of the workplace and the workers by ergonomists and experts who coach the workers or suggest the redesign of workplaces. This approach faces at least two challenges; the first is due to differences in workers’ individualities, e.g., height and body size. The solution to a risk factor, e.g., unforced demanding posture, may be to coach the workers towards a less demanding and less risk-inducing work technique. However, it is often difficult to change a habitual pattern of movement. In large organizations with manufacturing and assembly lines, regular inspection and coaching by ergonomists is often used to customize the workplace to the workers, e.g., providing the possibility for shorter persons to work in an assembly line. In cases where there is a need to modify a worker’s technique, changing is trickier. A few coaching sessions might appear to be sufficient to train the workers toward more ergonomic work behavior. However, people tend to go back to their old working habits, reducing the effect of these training efforts [12].

Direct measurement techniques provided by accelerometers, gyroscopes, magnetometers, electromyogram (EMG), and heart rate recorders have been used in ergonomics research since the nineties [13,14,15]. However, these systems are relatively expensive, complicated to use in the data collection and analysis phases, and have not been widely used by ergonomists and occupational healthcare providers. Electronic sensors, e.g., inertial measurement units (IMUs), have advanced dramatically during the past two decades, making them widely accessible, more affordable, and more compact in form. Additionally, wearable sensors, such as sensorized garments, smart watches, and wristbands, are changing the traditional interaction with users in welfare and healthcare. Wearable solutions may be a natural way to improve the usability of measurement systems by avoiding the use of cables and sticky electrodes and ensuring correct electrode placement by novice users [16].

The use of wearable sensing technologies enables continuous and long-term monitoring (i.e., full work days) for as many work days as needed, successively or at repeated time intervals (e.g., once a month) for a reliable assessment or successful work technique intervention. Long-term usage allows for a personalized coaching approach with continuous feedback and risk trend analysis. Informing people about their behavior and risks could be a first step toward behavioral changes, which can be supported with strategies such as gamification. Gamification is the use of gaming elements, e.g., points, badges, trophies, and awards, in a collaborative or competitive environment to support behavioral changes [17].

In this work, we present a platform that enables precise risk assessments and personalized automatized coaching. Because it is precise, pervasive, and personalized, we call it the P-Ergonomic platform. The platform is designed for producing a generic assessment and coaching by using in-house developed garments and a mobile application, as well as third party solutions. The platform has a generic base and may be used for other applications, e.g., in sports and medical kinetic training; the current application is focused on MSD prevention and changing sedentary work behavior.

In the following sections, the specification of the system and proposed architecture are described in detail, followed by the methods for technical validation. The results of the technical evaluation are presented in Section 5, followed by the discussion and conclusion.

## 2. System Specification

The main objective of the p-Ergonomics platform is to provide a reliable and flexible foundation for data collection, storage, analysis, and feedback to the end users, i.e., workers, employers, coaches, and occupational healthcare providers. The business cases for p-Ergonomics include small- to medium-sized businesses, as well as large enterprises. Since these businesses have different budgets and policies, the system architecture should consider the flexibility of deploying the platform at local or cloud servers using a cost-effective approach, which is scalable for big enterprises and affordable for small businesses, e.g., self-employed hairdressers or ergonomic coaches.

Security aspects are important features to ensure the privacy of workers. Authentication and authorization should be implemented to ensure accredited access to specific actions according to the user’s role and granted permissions. Action logs should provide the possibility of auditing the activities. The confidentiality and integrity of the data should be ensured with different measures, e.g., encryption. The security features should provide the possibility for compliance with restrictive data and information regulations, e.g., the General Data Protection Regulation (GDPR) [18].

The system should be flexible, which means a layered architecture to allow for changing different modules, e.g., sensors, third party integration, and analytics, without modifying the entire system. Considering the performance, a minimum required performance of 500 concurrent users for each deployed instance of the system should be tested for a target availability of 24/7.

The system should handle internationalization (i18n), providing support for i18n in the presentation layer and other layers, e.g., the analytic layer. Usability is another important requirement; the system should be easy to deploy, learn, and use by different stakeholders, such as occupational healthcare providers, administrators, and end users, i.e., workers.

## 3. Proposed Architecture

**Figure 1:**
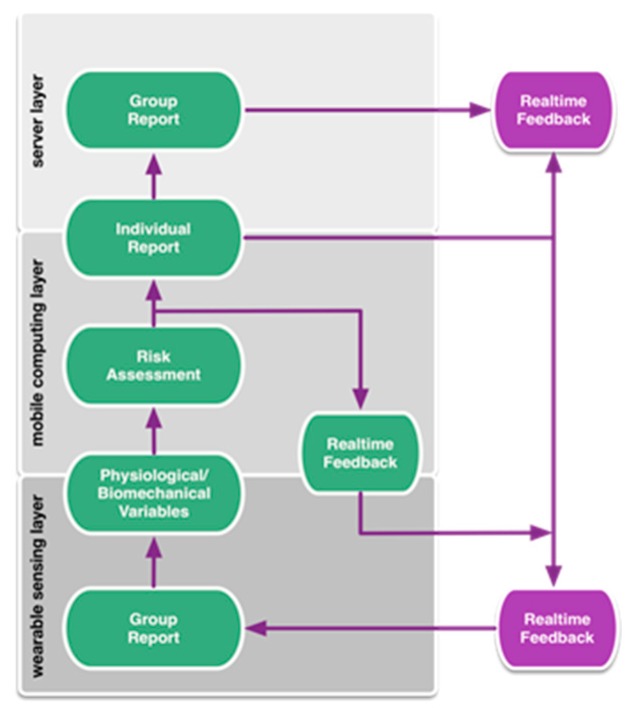
The proposed system architecture, hardware layers, and information flow.

The system collects data generated by wearable sensors, processes it for the purposes of risk assessment, intervention, and evaluation, and delivers the data to different stakeholders, including the worker, coach, and manager. The proposed system architecture is illustrated in Figure 1. The system consists of three layers: a wearable sensing layer, a mobile computing layer, and a server layer. The wearable sensing layer provides various physiological and biomechanical measurements and preprocesses the raw signals. At the mobile computing layer, data from each sensor node is collected, synchronized, and processed into variables reflecting exposure. The risk levels are then assessed according to defined ergonomic criteria and real-time feedback can be sent to the individual for intervention. Data from each individual is collected and stored at the server layer, where further analyses at both individual and group levels occurs. The server layer also employs user management for data logging and information access.

### 3.1. Wearable Sensing System

**Figure 2:**
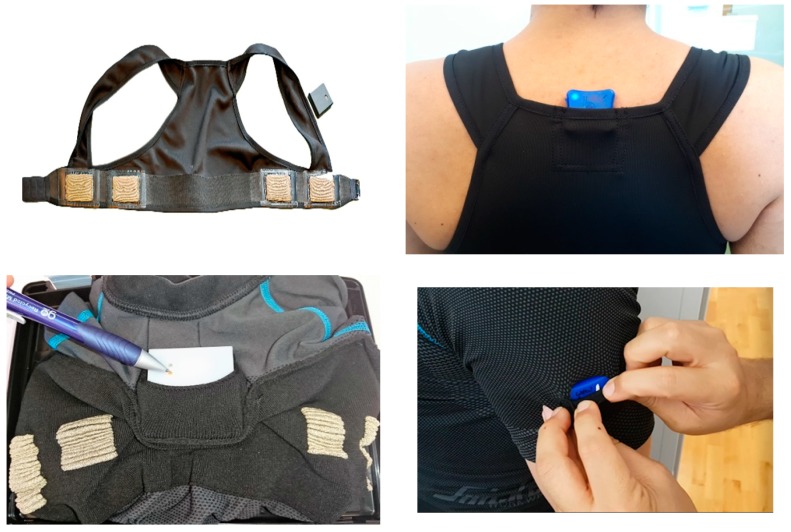
Vest (**top**) and t-shirt (**bottom**), including textrodes and ECGZ2 and IMUs.

The wearable sensor system consists of a t-shirt or vest, reported in other studies [19,20,21,22,23,24,25], which includes four textile electrodes made of conductive fabric. A pair of electrodes is used for the current injection and the other pair senses the electric potential. An ECGZ2 device (Z-Health Technologies AB, Borås, Sweden) was used as the recorder for electrocardiography (ECG) and electrical bioimpedance and was placed in a pocket on the shoulder strap of the vest or front of the t-shirt, as shown in Figure 2. ECG and thoracic impedance signals were recorded with sampling rates of 250 Hz and 100 Hz, respectively. One and two LPMS-B2 inertial measurement units (IMUs) (LP Research, Tokyo, Japan) were placed in the pocket on the back of the t-shirt or vest and t-shirt sleeves, respectively. The Polar A370 wristband smart watch (Polar Electro, Kempele, Finland) was included to monitor the daily activity and nightly sleep outside of working hours.

### 3.2. Personal Analytics and Coaching App

**Figure 3:**
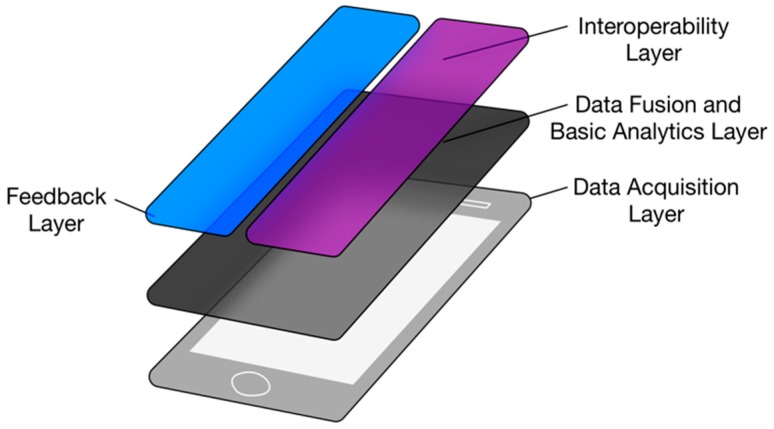
Distribution of the four layers that comprise the Edge node.

Analysis of the data from the different wearable sensors and the provision of feedback to the user is done through different software layers. The software runs on an edge-computing node in the form of a smartphone device with an Android operation system. Figure 3 illustrates the different layers of the system. This approach facilitates independence between layers, allowing for the update or substitution of any of them without hindering the system’s performance.

#### 3.2.1. Data Acquisition Layer

Data are acquired from the different wearable sensors, which make use of the Bluetooth communication standard to connect to the smartphone device, emulating a body area network operation. The user can select from a list of sensing devices based on the needs of different ergonomics variables of the assessment. The options are inertial measurement units (IMUs), and electrocardiogram (ECG) and thoracic bioimpedance recording devices. The smartphone connects to the sensors, initially configuring them based on preset specifications, emphasizing the frequency control of data acquisition and communication, filtering implementation, and data formatting.

This layer also controls and applies calibration algorithms, which require a direct interaction with the configuration functions of the sensing devices’ firmware, enabling treatment of the raw data from the sensors to calibrate the referential systems for inertial measurement units and other referential-type sensors.

This layer formats the acquired raw data to a standard format to be used by the subsequent layers. Synchronization of recordings from different sensors is done by adding timestamps to the data.

#### 3.2.2. Data Fusion and Basic Analytics Layer

This layer provides the system with the capability to transform the raw information extracted from the sensors into added-value information that can be analyzed to perform real-time multimodal risk assessments.

Firstly, preprocessing of the standardized raw data is performed. Data from the sensors is transformed from their original units, i.e., quaternions from IMUs or voltage from electrocardiographic recording devices, into units or information that are interpretable by the risk evaluation algorithms, i.e., transforming quaternions into Euler angles or voltage from electrocardiographic measurements into heart rate frequency. This process also includes data fusion from different sensors to produce additional useful information for the assessment, i.e., relative angles and angular velocity between two IMUs located in the body area network.

Processing and sensor fusion results in dramatic reduction of data sent to the cloud data warehouse. As an example, data from IMUs includes 3-axis gyroscope, a 3-axis accelerometer, and a 3-axis magnetometer sampled at sampling rates around 20−100 Hz depends on application. However, processed data from sensor fusion might just include the limb angles at a rate of 1 Hz sent to the cloud data warehouse. In some working scenarios, such as production lines, it might be interesting to look at workload at specific working or break cycles. To cover this scenarios, an extra processing of data can be done by allowing categorization of data based on sensor data, e.g., location data showing a station or even manual time stamps.

Finally, action policies are applied to enable the production of risk assessments based on the requirements established by the users and existing risk assessment algorithms, such as Risk Assessment and Management tool for manual handling Proactively (RAMP) screening [26].

#### 3.2.3. Feedback Layer

Given the possibility of estimating the risks, the real-time analysis of the measured parameters allows for the production of feedback in real time, based on the triggering of different events abiding to the different action policies chosen by the users. The feedback for this system prioritizes user-friendly notifications, descriptions, and instructions that are easily understood by different users.

Different types of feedback can be used to adapt to different scenarios. Visual feedback, such as the display of information through the graphical display of the smartphone, can provide different levels of information based on the user’s profile and display different parameters continuously, e.g., heart rate and activity recognition. Visual feedback could be a detailed report of the session or simply color-coded icons representing the different levels of risk, as well as a description of what caused that problem, enabling the users to minimize it.

The system is not limited to visual feedback. In most common working scenarios, the workers might not be able to interact with the screen, so an audio feedback system is preferred. A series of predefined coaching messages are given to the worker if an activity has been recognized as exceeding a risk threshold. The audio feedback is given at time intervals defined by the user. Haptic feedback using the actuators integrated in the wearables has also been tested and showed promising preliminary results.

#### 3.2.4. Interoperability Layer

This layer provides the body area network with the possibility of connecting to a remote server solution to send the information processed by the data analytics and fusion layer. By formatting the time-stamped processed data into a JavaScript Object Notation (JSON) message, it is possible to use the Hypertext Transfer Protocol (HTTP) Representational State Transfer (REST) service to upload the data to a cloud platform or to a local server, depending on the restrictions of the deployment environment. In this sense, this layer offer to users the possibility to specify the server that has implemented similar REST APIs, described in Table 1, Table 2 and Table 3.

By default, the accumulated data of the previous five minutes is uploaded, but the user can change this uploading speed to as fast as every second, allowing for the use of the information of the body area network for remote monitoring in a close-to-real-time manner. At the end of a work-day, the total work exposure is saved on the server, and a statistical summary may be generated, with variables that may be compared the recommended action levels, and to the levels of other occupations [27,28]. Especially when a group of workers have been using the system at the same workplace, this is a very time efficient way of objectively obtaining precise work exposure, and risk analyses, at a work group level.

### 3.3. Data Warehousing and Business Logic

The server was developed following a RESTful software architecture approach. This means all resources or services offered by the system on the server side are offered through HTTP methods. The data is stored in an open source relational database, PostgreSQL version 9.6.8, with a management layer by TimeScaleDB (Timescale Inc, NYC, US) added for the treatment of time-series records.

**Figure 4:**
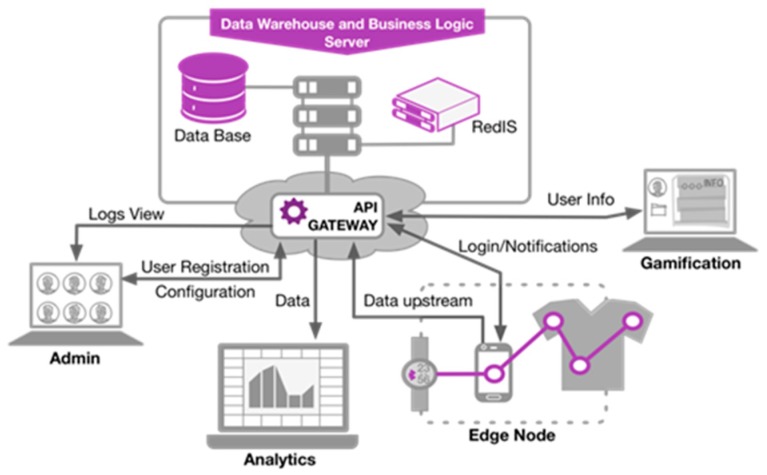
Data Warehouse and Business Logic Server overview and its interoperation with the other elements of the system.

The Node.js 10.13.0 programming language was used for implementation of the server. Due to its nature, Node.js is oriented to the development of web applications, facilitating the encapsulation of all functions and procedures of the server into small RESTful services. Through a RESTful API, these services are offered to the other components of the system as simple web resources, identified by a textual representation. Figure 4 shows an overview of the relationships between the server side solutions and the other elements through the RESTful API.

**Figure 5:**
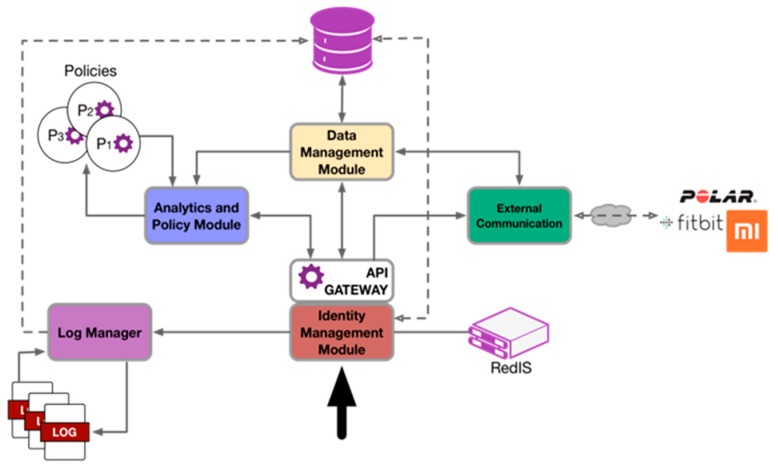
Distribution of the modules that comprise the analytical server and the interactive processes between them.

The internal design of the architecture is based on modules. Each module brings together the functions and resources related to a specific objective: identity and security management, log management, data management, communication with third-party applications, and management of action policies. Figure 5 shows an overview of the architecture and the interaction between modules. These interactions can be generated by the direct action of the user (solid arrow) or as a consequence of an internal process (dashed arrow). The following sections define each of the modules.

#### 3.3.1. Identity Management Module

The security management of the server is based on a simple access and identity management system. Secure communication is implemented through HTTPS channels between the clients and the server and the use of REDIS and PassportJS as a solution for the management of certificates, sessions, and cookies.

This module enables control of access to the resources offered by the server and management of the users. This module allows for the management of permissions to the resources to generate different user profiles. Thus, the system can discern and adapt the interactions according to whether it is an administrator user that is more focused on the management of the system, end users, whether they are information consumers (analysts and experts) or workers, and other profiles necessary for the correct deployment of the solution. The resources offered by this module are detailed in Table 1.

#### 3.3.2. Log Manager

All actions in the system must be logged to ensure the integrity of users. Therefore, the Log Manager registers all activities carried out in the server to process. The recorded logs are stored following the Common Log Format [29]. Through collaboration with the Data Management module, this module allows for exporting logs to a CSV format file that can be analyzed by experts. This functionality is encapsulated in a service managed by the Data Management module (see Table 4).

#### 3.3.3. Data Management Module

In general, the P-Ergonomics solution uses two types of data—those generated by wearable sensors, and those obtained from the evaluations of the user’s state, e.g., questionnaires or expert comments. In addition, sensors might be placed on different body parts or different types of questionnaires and subjective assessments can be used. Therefore, the data management module offers the resources required to support different data structures. The data of the sensors correspond to the structure described in Table 2. The information collected from the questionnaires is managed following the data structure detailed in Table 3. The resources offered by the RESTful API of the system related to the information management module are defined in Table 4.

#### 3.3.4. External Communication Module

The interactions between the system and external agents are managed through the external communication module. In the presented version, interaction with wearable solutions from Polar Electro, Kempele, Finland, through the Polar AccessLink is implemented to retrieve the relevant user information, e.g., sleep time, daily activity, consumed calories, and resting heart rate. This integration is done through an Open Authorization (OAuth) protocol by asking users to share their Polar information with this platform. The access token and user identifier in the third-party system, e.g., Polar, are kept for the future operations, e.g., obtaining data from AccessLink (https://www.polar.com/accesslink-api/#authorization-endpoint). The resources associated with this module are detailed in Table 5.

#### 3.3.5. Analysis and Action Policy Module

The server offers the possibility to manage action policies based on the data collected by the sensors and questionnaires. These actions are based on previously designed analysis libraries that are included in the system before execution. The analysis results of the user data are managed following the guidelines established in the action policies, i.e., store or send to the user or to the coach.

In addition to internal feedback and notifications, this module allows for sending messages to third party applications. In the current version, it interacts with Pocket mHealth (ATOS, S.A., Madrid, Spain) [30]. The management of notifications in this third-party application is beyond the scope of the article. The resources offered by this module are described in Table 6.

**Figure 6:**
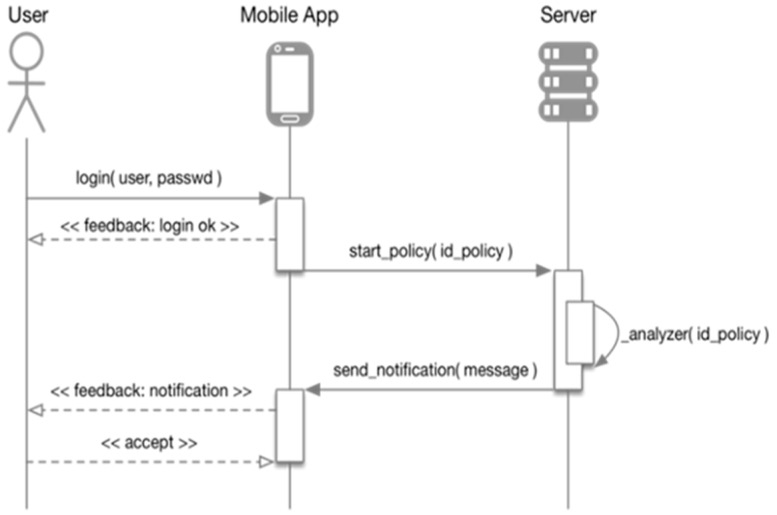
Sequence diagram of the action policy execution and interactions involved.

The process followed by this module to manage the action policies defined in the system and the interactions involved are shown in Figure 6.

## 4. System Validation

The proposed architecture and its implementation were validated through a realistic testing scenario. The objective of this validation was to evaluate the functionality, performance, and efficiency of the system for real deployment environments. Therefore, two types of tests were defined: a performance evaluation and a usability assessment.

### 4.1. Usability Assessment

The first stage of validation is to measure the usability of the system. A quantitative usability test, the System Usability Scale (SUS), was used [31]. The SUS questionnaire is a simple tool based on ten items to give a global view of subjective assessments of usability; that is, the effectiveness, the efficiency, and the user satisfaction related with the use of the system in order to perform a specific task. This test was carried out with N = 20 users in 4 groups of 5 users. As Nielsen noted in previous studies [32,33], this set of users is sufficient to tackle most usability problems and provides an overall view of the usability of the system. These users were selected from two different work types—office work and hospital work—to represent white- and pink-collar workers. The tests were carried out over 4–5 h for at least a working day in Spain and Sweden. In addition, all issues that occurred during the execution of the work activities were logged by an expert, such as interruption of the workflow due to system malfunctions. All participants were fully informed about the study and provided written consent. Ethical permission for the tests and data collection were obtained from the Regional Ethical Review Board in Stockholm (Dnr 2017/1586-31) and the local ethical board at Atos S.A.

### 4.2. Performance Evaluation

**Figure 7:**
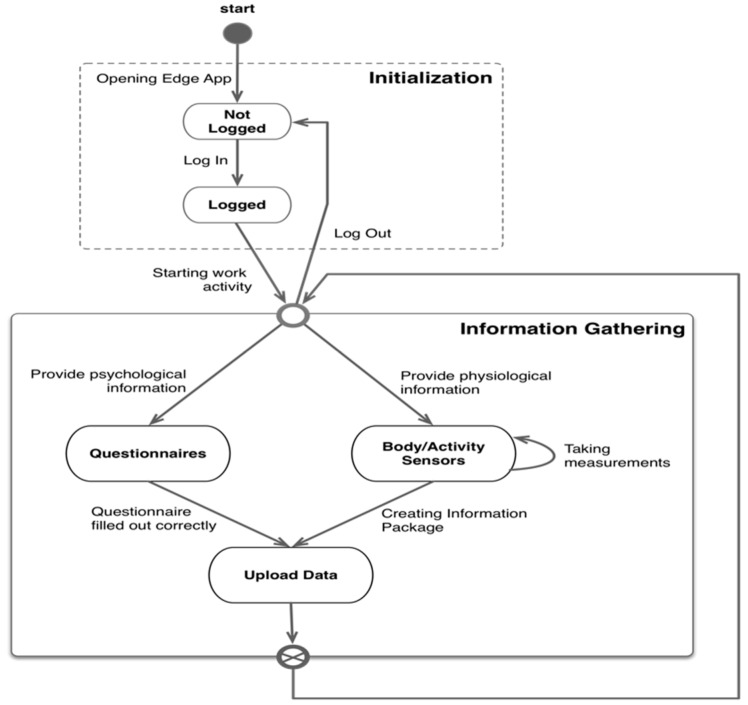
State diagram of common user activity with the Edge App.

Another important aspect is to test the functional viability of the system using a performance test. The design of this test is based on the common behavior of a user while interacting with the system, i.e., skipping the connection setup of the wearable sensors. This behavior model is shown as a state diagram defined in Figure 7.

Figure 7 shows the user interaction with the Edge App focuses on two main activities, the initialization or identification and the data collection. These two activities must be considered in the design of the performance test. Two processes comprise the information gathering, one related to the filling of questionnaires, and the other to the use of body sensors to measure movements and record physiological signals produced by the user. In both cases, the operation is similar: collect information, package it, and send it to the server to be stored and processed. However, the task of processing the data generated by the sensors is more complex due to the preprocessing performed in the Edge node. Therefore, the information gathering activity is modeled focusing only on the user’s interaction with the questionnaires, as it considerably simplifies the implementation of the test.

A Node.js library, Artillery.js, was used to implement the test using a human-readable data serialization file (YAML). This file defines two functional scenarios, where a virtual user should sign in and send the information related to a questionnaire to the server, with a similar workload to sending sensor data. Considering the probability of each scenario in real life, a weight was assigned to each, 1−4% to log in and log out, and 96−99% to upload information to the server.

As noted in previous research [34], a common scenario for a normal large company (logistics, manufacturing, etc.) is to have 120 workers for the same work line. However, a set of 50 simultaneous users could represent small-medium enterprises (SMEs) and 200 to 500 is more suitable for large corporations with more than 120 simultaneous users to be analyzed, if applicable. Thus, the test was performed by simulating 50, 120, 200, 300, and 500 users.

In terms of hardware, an Amazon Elastic Compute Cloud (Amazon EC2) (Amazon, Seattle, WA, USA) instance running the Ubuntu Server 16.04 was used to deploy the server, starting with the most restrictive set of resources, Amazon EC2 instance t2.nano. The test increased the resources, i.e., the Amazon EC2 instance type, when a use case demanded more CPU or memory. The client for data generation was simulated in an Amazon EC2 instance type t2.medium with Ubuntu Server 16.04, with similar hardware features to those offered by the real edge nodes used by the system. A description of the Amazon EC2 instances is presented in the Table A1.

## 5. Results and Discussion

**Figure 8:**
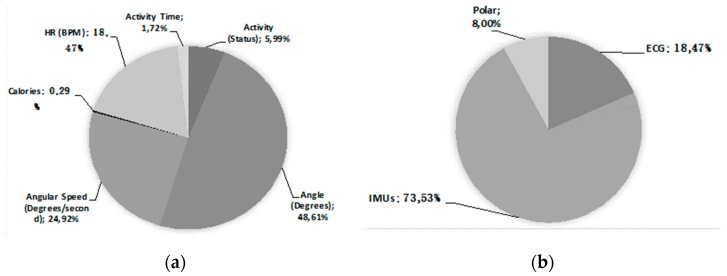
Distribution of the measures according to the type (**a**) and sensor that generated it (**b**).

The platform was tested in two different real work scenarios—one corresponding to white-collar workers at the ATOS offices in Madrid, Spain, and the other corresponding to pink-collar workers at the sterilization unit of the New Karolinska University Hospital in Stockholm, Sweden. Twenty users were enrolled and the system was tested for 4–5 h during a workday. These trials generated 2,601,016 measurements, an average of 130,050.8 per user. These measurements were used and analyzed to determine new activity detection algorithms, define new ergonomic suitability comparisons between jobs, or evaluate new occupational risks. Figure 8 shows the classification of the stored measurements and how each sensor contributed to the total. The IMU sensors (3 in each T-shirt) generated 73.53% of the measurements stored, with the angle between them being the most common type of measurement (48.61%). The least information came from third-party devices (Polar) at 8% of the total, because the raw data is stored in the Polar Access link server and only the essential information for the ergonomic analysis is retrieved, processed, and filtered according to the needs of the final application, i.e., calories, activity levels, and active steps.

**Figure 9:**
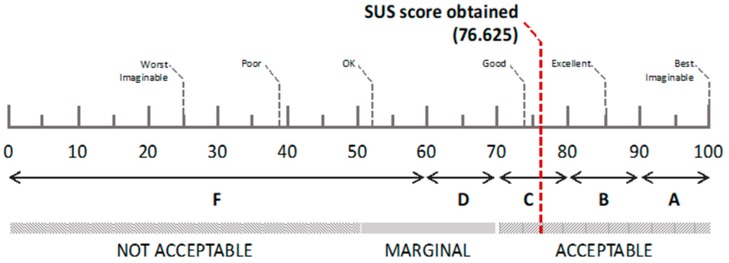
The SUS score of the system (dashed red line), with a visual explanation of adjective ratings, acceptability scores, and school grade scales in relation to the average SUS score [35].

The analysis of the answers obtained from the usability tests (N = 20) yields an SUS score of 75,625 out of 100. According to most interpretations [35], this score indicates that the system has an acceptable usability level, i.e., a C grade. However, as shown in Figure 9, there is room for improvement.

**Figure 10:**
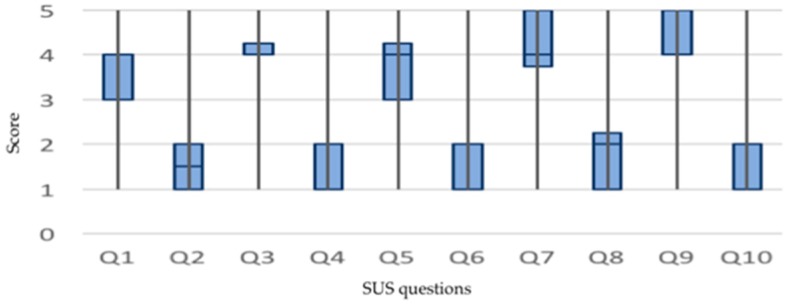
Graphic representation of the scores given by the users to each question of the SUS questionnaire. The questions are detailed in the Table A2.

This margin of improvement is visualized in the representation of the answers given to each question of the questionnaire outlined in Figure 10.

Note that the even questions refer to positive aspects of the usability of the system and the odd questions refer to negative aspects. It has observed that the system must improve around questions 1, 5, and 8. Question 1 refers to the user’s desire to use the system at all times. In this sense, the use of wearable elements and smart clothes appears to condition the final acceptance due to comfort aspects and doubts related to cleaning. Question 5 refers to the level of integration of the elements that comprise the system, that is, the functional coherence between the sensorized elements, such as the smart shirt, watch, and app. Question 8 refers to the comfort of the system, i.e., the wearable elements, which must be revised to increase the overall comfort of the system. The questions are detailed in the Table A2.

The performance test results are shown in Table 7. Depending on the number of users, it is possible to select a more restrictive Amazon EC2 instance type. The cost of each option is estimated by the amount of CPU credits used by the instance in 24 h (see Table A1 for the amount of credits offered for each instance type). The minimum amount of CPU credits necessary for a workday were estimated for each test. It is worth mentioning that performance numbers reported in Table 7 are heavily dependent on software implementation and cannot be generalized.

The performance scenario is designed as a worst case scenario, with simulation of request to the server at every second. However, in our actual scenario, one-second interval warehousing and cloud analysis of data is not required. Each limb data, from IMUs, was 150 bytes, and hence size of each packet corresponds to Header Bytes + (number of seconds * (number of sensors * 150)). In our pilots, we have used 300 s for synchronizing with the data warehouse, while the performance simulations are done in one second. Perhaps it might be desired to change to a binary communication in future to increase the efficacy.

**Figure 11:**
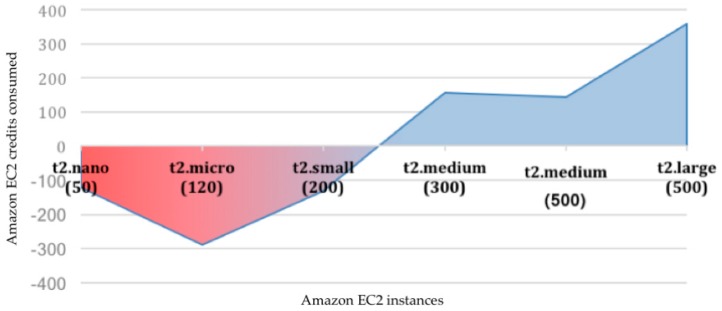
Graphical representation of difference between Amazon EC2 CPU credits offered by upfront payment for reserved Linux instances (type t2).

Figure 11 shows the difference between the minimum credits needed and those offered by each type of Amazon EC2 instance on dedicated Linux servers. Although the most affordable instances (t2.nano, t2.micro, and t2.small) offer adequate performance for few users, the final price can be altered due to the number of CPU credits used. In contrast, an instance of type t2.medium offers the best performance/credits-used ratio. With an annual advanced payment of $312, it is possible to handle up to 500 concurrent users with a CPU usage of 52%.

Another requirement of the system specification is the possibility to deploy the system on a local server. This is suitable for enterprises with highly restrictive policies about storing data on the cloud. The analytical part of the system, although it is implemented following a cloud service, can be deployed on private local servers. To achieve optimal performance similar to that offered by an Amazon EC2 t2.medium instance, the server specification is at least a dual-core processor and 4 GB of memory. Servers with such a specification are very affordable.

The development of the P-Ergonomics platform is in line with projects by our research group at the Royal Institute of Technology (KTH) and Karolinska Institutet, Sweden, and by our research partners. It is a part of the European Institute of Innovation and Technology (EIT) Health funded project [23], We@Work (http://weatwork.eu/). In a previous study [24], we demonstrated a wearable system integrating textrodes, motion sensors, and real-time data processing through a mobile application. Heart rate, respiration, and motion measurements obtained from a wearable system were fused to enhance the energy expenditure estimation [36,37]. Preliminary results of changing workers’ behavior after giving feedback is also reported [38]. The aim is to develop a comprehensive solution to promote and support a healthy and safe working life. To the authors knowledge, this approach is novel and has not been reported elsewhere.

## 6. Conclusions and Future Work

The developed platform shows promising results in collecting data from the edge node (Android application). For its part, the functional analysis has been done with a specific platform (Amazon EC2) and different results could be achieved with other platforms, such as Google Cloud or Microsoft Azure. In addition, the performance and capacity could be expanded by software or operating system optimization, addition of hardware resources, or the detailed study of communication and data exchange between the node and the server, i.e., data compression algorithms. However, the results show that as a whole, the capacity and affordability of the implemented system meets the required specification.

Inclusion of protocols that make use of universal sensors is another possible expansion of the system that can increase its versatility. The usability tests show acceptable results, although the use of specific smart clothes seems to condition the final acceptance. This has caused the redesign of a new integration of the ECGZ2 device in the wearable garment by its integration into the t-shirt directly, avoiding the use of the vest. Thus, more detailed tests, including long-term usage of the new wearable systems, are planned for future work. A detailed test of the system for changing users’ behavior by giving relevant feedback is ongoing. The use of gamification for engagement of workers is already planned and will be implemented. The use of pervasive and wearable technology in ergonomics could be a vital factor in enabling cost-effective ergonomic risk assessments and solutions in the near future.

## Figures and Tables

**Table 1:** Resources provided by the Identity Manager Module.

Resource	Method	Description
/auth/login	POST	Performs the login in the system. As a response, the system sends an authentication token.
/auth/register	POST	Registers new simple users in the system.
/auth/logout	GET	Log out from the system.
/auth/status	GET	Obtain the current status of the user and it informs users if they are logged or not.
/auth/regdevice	PUT	Register a new mobile device in the system. This mobile is syndicated to the user (authenticated previously) that uses this resource. See Section 3.3.3.

**Table 2:** Data Structure of a Sensory Measurement.

Data Field	Description	Value Example
tstamp	Time stamp when the data is stored into the system.	*2018-07-23 12:37:37.206017+00*
type	Short description or type of the measurement, i.e., calories, sleep time, etc.	*Angle (Degrees)*
position	Location of the sensor, if relevant, such as left arm, right wrist, back, etc.	*Back/Trunk*
sensor	Sensor type, i.e., IMUs, Polar watch, etc.	*LPMS-B2 IMU*
value	Final value stored in the system and its interpretation, conditioned by the type of data (type field).	*−3.63287*
id_user	User from whom the measure is taken.	*6*

**Table 3:** Data Structure of a Questionnaire Response.

Data Field	Description	Value Example
id_response	Unique identification of the stored response.	*123*
id_user	User who completed the form.	*14*
response_tstamp	Time stamp when the response is stored into the system.	*2018-07-23 12:37:37.206017+00*
response	Response structure, e.g., 5#4#5#2#2#3#1.	*4#4#5#2#1#-#3#3#-#1#2#1*
q_type	Type of form, indicating the policy to be applied for its analysis.	*Stress-Energy*

**Table 4:** Resources provided by the Data Management Module.

Resource	Method	Description
/api/v1/data/logs	GET	Obtain the logs of the system in CSV format.
/api/v1/data/msr	GET	Obtain all measures of all users. Only administrators can perform this action.
/api/v1/data/msr	POST	Store measurements in the database. This resource admits a set of several measurements or a single one.
/api/v1/data/msr/{id}	GET	Obtain all responses stored in the system. Only administrators can perform this action.
/api/v1/data/qtn	GET	Obtain the current status of the user, i.e., this resource informs users if they are logged or not.
/api/v1/data/qtn	POST	Store responses of a questionnaire in the database. This resource admits a set of several measurements or a single one.

**Table 5:** Resources provided by the External Communication Module.

Resource	Method	Description
/external/polar/auth	GET	Connect with Polar AccessLink API and start the authentication process.
/external/polar/callback	GET	Stores authentication credential (access token and user identification) of the user from the Polar AccessLink API.
/external/polar/register/{id}	GET	Registers a worker in the Polar AccessLink system. This action is mandatory to allow users to access their data.
/external/polar/delete	GET	Revokes the authorized access token provided by Polar.
/external/polar/listOf/{performance}	GET	Access and process the user’s daily activity data from the Polar AccessLink and store them in the system database.

**Table 6:** Resources provided by the Analysis and Action Policy Module.

Resource	Method	Description
/api/v1/notification/pmh/{msg}	GET	Send notifications and messages (msg) to external services. The system uses an external notification API based on Firebase [30].
/api/v1/notification/pmh	GET	Perform analysis related to the health at work policy associate. The policy is defined as a main function in a library.

**Table 7:** Summary of the performance test results.

Amazon EC2 Instance	User/sec	CPU Usage	Amazon EC2 Credits Used	Average Latency (ms)
T2.nano	50	37%	1.6	3.5 (p95 = 174.6)
T2.micro	120	72%	3.6	93.7 (p95 = 548.5)
T2.small	200	72%	3.5	61.3 (p95 = 420.7)
T2.medium	300	42%	3.5	75.2 (p95 = 468.8)
T2.medium	500	52%	3.6	154.7 (p95 = 658.8)
T2.large	500	59%	4.2	591.8 (p95 = 2485.7)
T2.nano	50	37%	1.6	3.5 (p95 = 174.6)

## Appendix A

The Table A1 presents a summary of the features offered by Amazon for using Amazon EC2 Virtual Machines. This summary is only focused on the T2 Amazon EC2 instance, the most affordable modality, which is ideal for a variety of general purpose applications. In addition, the description of the T2 instances has been limited only to those used in the test, i.e., nano, micro, small, medium, and large.

**Table A1:** Characteristics of the Amazon EC2 instances.

Amazon EC2 instance	CPUs	Credits/hour	Memory (GiB)	Upfront Price ($)
T2.nano	1	3	0.5	39.00
T2.micro	1	6	1	77.00
T2.small	1	12	2	156.00
T2.medium	2	24	4	312.00
T2.large	2	36	8	625.00

## Appendix B

The usability analysis has been carried out through the use of the System Usability Scale (SUS). For this, the tested and accepted questionnaire that provides this method has been used. Table A2 specifies the 10 questions that make up this questionnaire.

**Table A2:** SUS questionnaire.

Item	Question
Q1	I think that I would like to use this system frequently.
Q2	I found the system unnecessarily complex.
Q3	I thought the system was easy to use.
Q4	I think that I would need the support of a technical person to be able to use this system.
Q5	I found the various functions in this system were well integrated.
Q6	I thought there was too much inconsistency in this system.
Q7	I would imagine that most people would learn to use this system very quickly.
Q8	I found the system very cumbersome to use.
Q9	I felt very confident using the system.
Q10	I needed to learn a lot of things before I could get going with this system.
